# Concurrent validity of the Fitbit for assessing sedentary behavior and moderate-to-vigorous physical activity

**DOI:** 10.1186/s12874-019-0668-1

**Published:** 2019-02-07

**Authors:** Nicklaus Redenius, Youngwon Kim, Wonwoo Byun

**Affiliations:** 1 Department of Health, Nutrition, and Exercise Sciences, North Dakota State University, Fargo, ND 58108 USA; 2 Department of Health, Kinesiology, and Recreation, University of Utah, Salt Lake City, UT 84112 USA; 3 Division of Kinesiology, School of Public Health, The University of Hong Kong Li Ka Shing Faculty of Medicine, Room 301D 3/F, Jockey Club Building for Interdisciplinary Research, 5 Sassoon Road, Pokfulam, Hong Kong; 4 MRC Epidemiology Unit, University of Cambridge School of Medicine, Cambridge, Cambridgeshire UK

**Keywords:** Physical activity, Sedentary behavior, Validity, Accelerometer, Public health

## Abstract

**Background:**

Recent advances in sensor technologies have promoted the use of consumer-based accelerometers such as Fitbit Flex in epidemiological and clinical research; however, the validity of the Fitbit Flex in measuring sedentary behavior (SED) and physical activity (PA) has not been fully determined against previously validated research-grade accelerometers such as ActiGraph GT3X+. Therefore, the purpose of this study was to examine the concurrent validity of the Fitbit Flex against ActiGraph GT3X+ in a free-living condition.

**Methods:**

A total of 65 participants (age: M = 42, SD = 14 years, female: 72%) each wore a Fitbit Flex and GT3X+ for seven consecutive days. After excluding sleep and non-wear time, time spent (min/day) in SED and moderate-to-vigorous PA (MVPA) were estimated using various cut-points for GT3X+ and brand-specific algorithms for Fitbit, respectively. Repeated measures one-way ANOVA and mean absolute percent errors (MAPE) served to examine differences and measurement errors in SED and MVPA estimates between Fitbit Flex and GT3X+, respectively. Pearson and Spearman correlations and Bland-Altman (BA) plots were used to evaluate the association and potential systematic bias between Fitbit Flex and GT3X+. PROC MIXED procedure in SAS was used to examine the equivalence (i.e., the 90% confidence interval with ±10% equivalence zone) between the devices.

**Results:**

Fitbit Flex produced similar SED and low MAPE (mean difference [MD] = 37 min/day, *P* = .21, MAPE = 6.8%), but significantly higher MVPA and relatively large MAPE (MD = 59–77 min/day, *P* < .0001, MAPE = 56.6–74.3%) compared with the estimates from GT3X+ using three different cut-points. The correlations between Fitbit Flex and GT3X+ were consistently higher for SED (r = 0.90, ρ = 0.86, *P* < .01), but weaker for MVPA (r = 0.65–0.76, ρ = 0.69–0.79, *P* < .01). BA plots revealed that there is no apparent bias in estimating SED.

**Conclusion:**

In comparison with the GT3X+ accelerometer, the Fitbit Flex provided comparatively accurate estimates of SED, but the Fitbit Flex overestimated MVPA under free-living conditions. Future investigations using the Fitbit Flex should be aware of present findings.

## Background

Surveillance of physical activity (PA) is vital for better understanding the relationship between PA and specific health outcomes such as obesity, hypertension, and type 2 diabetes. Although limitations of self-report are evident, historically surveillance data have relied on subjective measures of PA such as self-reported questionnaires [1, 2]. Accelerometers are particularly appealing for PA monitoring in free-living conditions; several accelerometer-based devices have been used in PA research applications [3]. ActiGraph accelerometer is the most widely used to measure PA in research and surveillance systems [3, 4]. For example, due to its high validity and reliability [5–8], the ActiGraph GT3X+ (GT3X+) was the method of choice for measuring PA in many population-based epidemiological studies [9–12].

Researchers have used the strong relationships between accelerometer and indirect calorimetry (IC) output to establish count-based prediction models for PA outcomes such as minutes of PA in metabolic equivalent of task (MET) categories or energy expenditure (EE) [13, 14]. Using regression equations with accelerometer counts (i.e. counts/60 s) as a predictor, several activity count cut-points have been developed to estimate the amount of time spent in sedentary behavior (SED) as well as different intensities of PA, namely moderate-to-vigorous intensity PA (MVPA). Two sets of cut-points developed by Freedson et al., one using single axis data (Freedson) and the other vector magnitude data (VM3), and one set developed by Troiano et al. are widely utilized cut-points for estimating time spent in SED and varying intensities of PA including, light intensity PA and MVPA [13, 15, 16]. The cut-points defined SED and MVPA as follows: 1) < 100 counts and ≥ 1952 counts/60-s, 2) < 100 counts and ≥ 2020 counts/60-s, and 3) ≥2691 counts/60-s (for MVPA only) for the Freedson, Troiano, and VM3 cut-points respectively.

However, PA intensity estimates may significantly vary depending on cut-point criteria applied to accelerometer output, which is primarily caused by inconsistency in monitor placement and types of activities used to calibrate the cut-points [17, 18]. As such, there is no single cut-point criteria able to accurately classify accelerometer-based PA estimates across all intensity categories and activities [17]. Thus, studies investigating the validity of PA monitors with an accelerometer-based criterion may be limited by utilizing only one cut-point prediction model. Nonetheless, the Freedson cut-points have produced acceptable estimates of MVPA even when compared to more recent cut-points [15, 17, 18].

Fitbit, Inc. (San Francisco, CA) is a leading manufacturer of accelerometer-based PA monitors sold in the consumer electronics [19]. Fitbit has achieved the largest market share among numerous wearable activity trackers available on the market [20, 21]. Given the popularity of these consumer-based PA monitors, there may be an opportunity to use them as a research tool. Few studies have examined the validity of Fitbit monitors, but most used only hip-worn Fitbit models (i.e. Fitbit Ultra, Zip, One) in controlled settings [22, 23]. Fitbit Ultra and Fitbit One step estimates have demonstrated strong agreement with directly observed step counts across a wide range of walking speeds in both adult and elderly populations [24–26]. Group-level Fitbit (i.e. Fitbit Ultra, Zip, One) EE estimates have demonstrated strong correlations with IC-determined EE, but correlations were lower at the individual-level [22, 23]. An investigation of the accuracy of the hip-worn Fitbit “classic” suggest it overestimates EE for cycling, inclined treadmill walking, stair climbing, and chores such as laundry and yard raking, whereas it underestimated EE of walking while carrying an external load (i.e. groceries) [27]. However, the sum of overestimates and underestimates of individual activities may lend hip-worn Fitbit monitors to provide reasonably accurate PA estimates over a variety of activities. The previous study by Lee et al. demonstrated that hip-worn Fitbit Zip yields EE estimates within 10% of IC criterion during a 69-min protocol of 13 different activities, including over ground walking, treadmill walking and jogging, playing basketball or active video games, etc. [23]. Few studies have evaluated the accuracy of Fitbit’s wrist-worn PA monitors, namely the Fitbit Flex. Whereas Fitbit Flex underestimates steps compared to direct observation evidence suggests it overestimates EE compared to IC criterion during treadmill walking and jogging [28–30]. Similarly contrasting results exist regarding Fitbit Flex PA estimates while performing common household activities. One study found Fitbit Flex overestimates steps while folding laundry and playing a game on a tablet whereas a different study found no significant differences in step estimates compared to direct observation as well as EE estimates (IC criterion) for other household activities (e.g. sweeping, standing, picking-up items) [29, 30]. As with many PA monitors, Fitbit Flex may over- or underestimate PA for certain individual activities while yielding reasonably accurate daily PA estimates. However, in a lab-based protocol of treadmill walking or running and resistance training exercises using machines the combined over- and underestimates of Fitbit Flex EE estimates did not fall within 10% of IC-determined EE [28]. Therefore, the purpose of this study was to examine the concurrent validity of the wrist-worn Fitbit Flex compared to the hip-worn ActiGraph GT3X+ utilizing three different cut-point criteria in a free-living condition.

## Methods

### Participants

A convenience sample of 65 participants (age: 41.7 ± 14.3 years, Female: 72.3%) was recruited via email, posted fliers, and word-of-mouth. Participants who were under the age of 18, pregnant, physically disabled, or unable to engage in regular PA as recommended by a physician, were not eligible to be in the study. The North Dakota State University Institutional Review Board approved the study and all participants voluntarily provided consent to participate in the investigation.

### Instruments

ActiGraph (ActiGraph Corp., Pensacola, FL) currently offers multiple models of tri-axial accelerometer-based devices. The ActiGraph GT3X+ is a lightweight (19 g), tri-axial accelerometer-based device with a dynamic range of −/+ 6 G. Users may choose sampling frequencies from 30 Hz to 100 Hz. We chose a sampling rate of 30 Hz (with one-minute epochs) as this range should adequately capture most accelerations due to human movement [29]. Data from GT3X+ accelerometer were downloaded and scored using ActiLife version 6.11.4 (ActiGraph Corp., Pensacola, FL).

The Fitbit Flex (Fitbit, Inc., San Francisco, CA), is a physical activity monitor that is 3.2 cm long and weighs less than 15 g (including wristband). It features a tri-axial accelerometer and continually acquires data and with onboard storage capacity for approximately seven days of data without syncing. Data is transferred via Bluetooth technology to the Fitbit application program interface (API) either through Fitbit’s mobile app or a Bluetooth dongle connected to a computer.

### Procedures

Participants completed an orientation session and began the free-living protocol after voluntarily consenting to be in the study and completing a demographic questionnaire. Participants were instructed to simultaneously wear the Fitbit Flex and GT3X+ monitors for seven consecutive days during all waking and sleep hours except during bathing and recreational water activities (e.g. swimming). Participants wore a Fitbit Flex monitor on the dorsal aspect of the non-dominant wrist, similar to a watch, which is a standard placement site for PA measurement using wrist-worn accelerometer as well as in compliance with the manufacturer’s recommendation [9, 31, 32]. The GT3X+ monitor was worn on the dominant hip in-line with the midline of the thigh and the approximate peak of the iliac crest, which has been known as a standard placement site of the GT3X+ [7, 33–36]. Participants kept a log of any non-wear time during waking hours and daily sleep times. Participants were also instructed to note any days that included extraordinary amounts of PA that may appear unusually high for their typical routine (e.g. running a half-marathon). Data from activity/sleep logs and activity monitors were retrieved and downloaded respectively at the conclusion of the data collection period. All the GT3X+ and the Fitbit flex monitors used in this study were not donated by the manufacturers, but purchased by the investigative team using our own research funding.

### Data reduction

Data from the GT3X+ was downloaded and converted into activity counts per 60-s epoch using the ActiLife software. The GT3X + ‘s activity counts data was then scored into daily time spent in SED and MVPA (min/day) by applying three different sets of cut-points: 1) Freedson [13], 2) Troiano [37], and VM3 (MVPA cut-point only) [5].

Since the Fitbit dashboard provides limited resolution of PA estimates (i.e., hourly summary), we chose to use a third-party research application program interface (API) called Fitabase (Small Steps Labs, LLC, San Diego, CA), which allows exporting the data from the Fitbit at 60-s sampling intervals. Unlike the GT3X+ accelerometer, using its proprietary algorithm, the Fitbit Flex converts raw acceleration data into activity counts in 60-s sampling intervals that define activity intensities as 0 = sedentary, 1 = light PA, 2 = moderate PA, and 3 = vigorous PA.

For the GT3X+, non-wear time and sleep time were defined using an algorithm developed by Choi et al. [38] and participants’ activity/sleep logs, respectively, and were excluded for further analysis. No participants noted any extraordinary PA during the study period. Similarly, Fitbit Flex wear time was validated by removing sleep and non-wear time from the participant activity/sleep log. After non-wear time validation procedures, minute-by-minute data from the GT3X+ and the Fitbit Flex were temporally aligned and merged into a single dataset, thus only valid wear time during waking hours that simultaneously recorded on both devices were included for statistical analysis.

### Statistical analysis

Pearson’s and Spearman’s correlations were used to determine the relationship between PA and SED estimates from Fitbit Flex and those from GT3X+. Due to unequal variances, we used the Welch’s T-test to assess differences in daily PA and SED within gender (male and female) and BMI category (normal, overweight, and obese), respectively. To avoid committing a Type-I error with SED and MVPA comparisons, repeated measures one-way analysis of variance (ANOVA) served to examine differences in SED and MVPA estimates, comparing Fitbit Flex estimates and those from GTX+ using three different cut-points (only two cut-points used for SED comparisons). Significant overall ANOVA effects were followed by pairwise comparisons using Bonferroni adjustment. Mean absolute percent errors (MAPEs) were calculated to assess similarity of the estimates from the Fitbit Flex in comparison with the GT3X+. Bland-Altman (BA) plots were used to illustrate the agreement between the GT3X+ and the Fitbit Flex as well as evaluate any potential random biases in SED and MVPA estimates between two devices. Pitman’s Tests difference in variance were performed to determine the equality of the variances in SED and MVPA estimates between two devices. Equivalence tests were performed to determine the agreement between the GT3X+ and the Fitbit Flex. The specified equivalence zone (EZ) was defined as ±10% of the mean estimates from the GT3X+ and compared with the 90% confidence intervals (CI) of the estimates from the Fitbit Flex. The estimates from the GT3X+ and the Fitbit Flex are equivalent if the CIs of the Fitbit Flex completely fall within the equivalence zone. All data analyses were conducted using IBM SPSS 24.0 for Windows (SPSS, Armonk, NY) and the SAS statistical program, version 9.4 (SAS Institute, Cary, NC, USA). Alpha level of 0.05 was set to define significance for all statistical analyses.

## Results

Subject characteristics are summarized in Table 1. The sample was relatively homogenous, mostly female, and non-Hispanic white. Participant ages and BMI ranged 20–70 years and 18.6–40, respectively. Daily minutes of MVPA and SED were presented separately for males and females (Table 2). Participants recorded an average of 5.8 valid wear days (14.9 h/day after removing sleep period) over the 7-day period. Participants spent the majority of waking hours in SED and least amount of waking hours in MVPA. We chose not to conduct a two-way multivariate analysis of variance (MANOVA) with sex and BMI as fixed factors due to lack of power to detect a difference due to a low number of participants in each stratum. However, Welch’s T-test results showed mean daily MVPA and SED did not significantly differ between males and females (Table 2). Similarly, we assessed differences in MVPA and SED between BMI categories (i.e. normal, overweight, and obese). Because there were few obese participants (*n* = 9), we combined the obese and overweight categories to avoid underpowered analysis. Welch’s T-test showed no significant differences in SED or MVPA between normal and overweight/obese groups (Table 2). Since no significant differences in MVPA and SED were observed between gender and BMI groups, and these comparisons were not integral to the intended analysis, we combined data from the entire sample (*n* = 65) for the remainder of the analysis.

**Table 1:** Participant characteristics by gender, Mean ± SD or percent

	Total (*N* = 65)	Male (*N* = 18)	Female (*N* = 47)
Age (years)	41.7 ± 14.3	36.2 ± 15.2	43.8 ± 13.5
Race (%)			
White	97.0	100.0	95.9
Other	3.0	0	4.1
BMI (kg/cm^2^)	25.9 ± 4.5	26.3 ± 4.0	25.8 ± 4.6
Weight Status (%)			
Normal	46.1	33.3	51.1
Overweight	40.0	55.6	34.0
Obese	13.9	11.1	14.9

**Table 2:** Mean valid wear days and mean daily minutes of MVPA and SED by gender and weight status, Mean ± SD

	Total (N = 65)	Male (N = 18)	Female (N = 47)	*P*-value^†^	Normal (*N* = 30)	Overweight/Obese (*N* = 35)	*P* -value^‡^
Valid Wear Days	5.8 ± 1.2	5.7 ± 1.2	5.9 ± 1.3	0.61	6.1 ± 1.3	5.7 ± 1.15	0.20
SED^a^ (min/day)							
Fitbit	637.8 ± 89.7	633.2 ± 85.5	639.5 ± 92.2	0.90	636.0 ± 79.0	634.8 ± 102.5	0.95
GT3X+ (Freedson/Troiano)	600.4 ± 92.4	623.1 ± 91.1	591.7 ± 92.4	0.22	595.5 ± 86.1	603.0 ± 97.2	0.73
MVPA^b^ (min/day)							
Fitbit	107.8 ± 32.1	112.6 ± 36.0	105.9 ± 30.7	0.44	105.9 ± 29.8	109.4 ± 34.3	0.66
GT3X+ (Freedson)	30.1 ± 18.5	29.3 ± 19.6	30.4 ± 18.2	0.91	31.2 ± 19.3	29.2 ± 17.9	0.66
GT3X+ (Troiano)	28.7 ± 18.1	27.9 ± 19.2	28.9 ± 17.8	0.91	29.9 ± 19.2	27.6 ± 17.3	0.62
GT3X+ (VM3)^§^	48.1 ± 24.2	45.5 ± 24.1	49.1 ± 24.4	0.69	46.2 ± 21.4	49.8 ± 26.5	0.54

^§^VM3: sedentary behavior estimates were not available from the VM3 cut-points

^a^SED: sedentary behavior; ^b^MVPA: moderate-to-vigorous physical activity

**Fig. 1:**
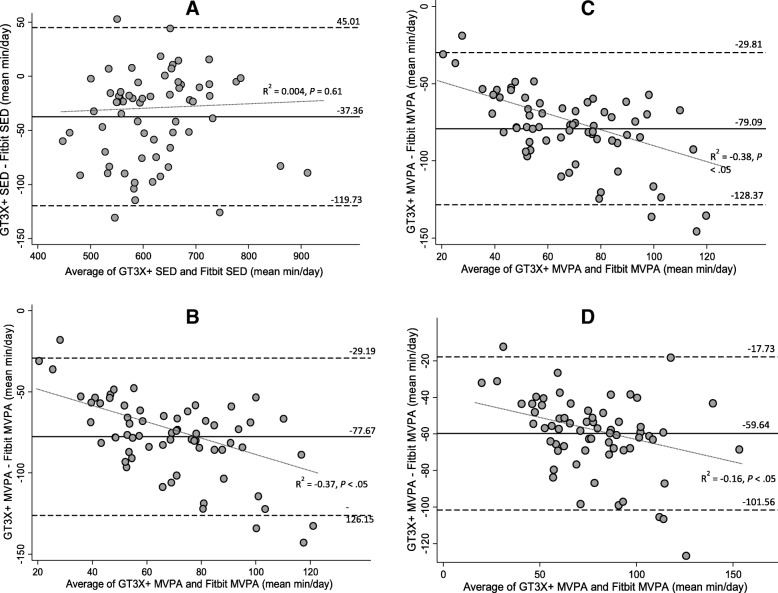
Bland-Altman plots illustrating level of agreement between GT3X+ and Fitbit. **a**) SED estimates (< 100 cpm); **b**) MVPA estimates (Freedson cut-points); **c**) MVPA estimates (Troiano cut-points); **d**) MVPA estimates (VM3 cut-points); Dashed lines show 95% limits of agreement (± 1.96 SD)

**Fig. 2:**
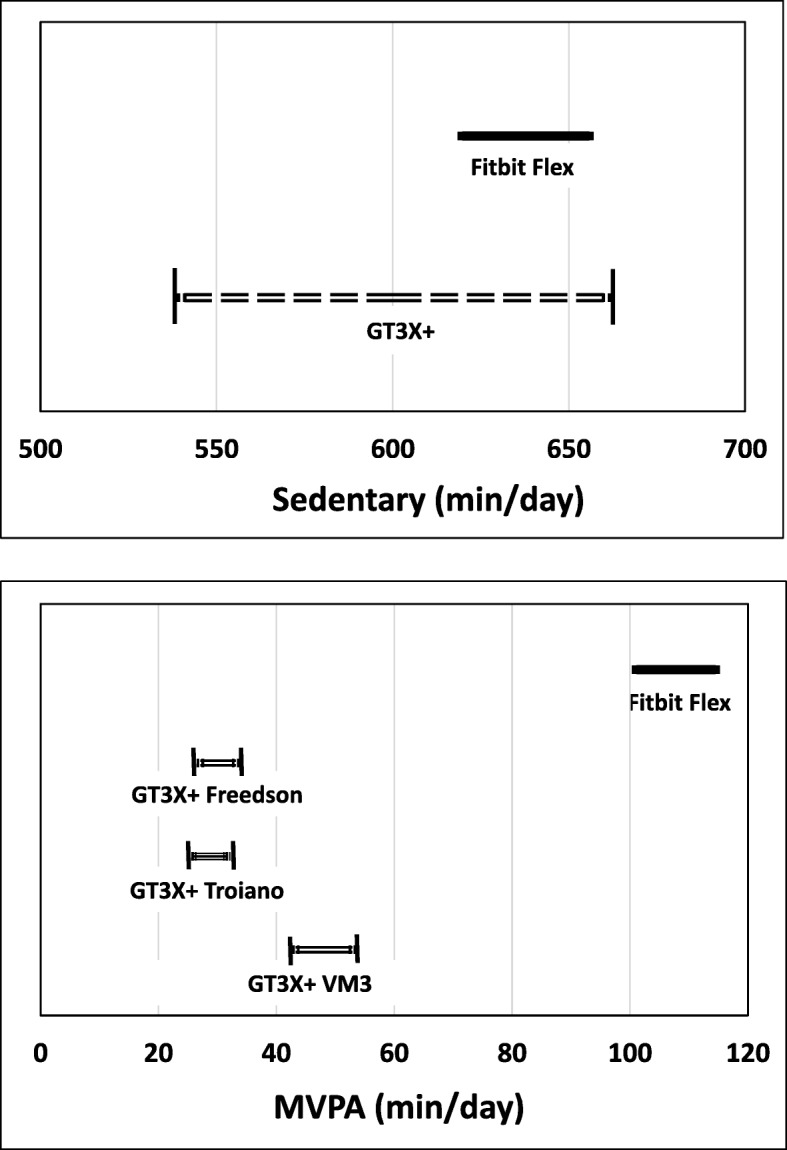
Equivalence Testing for SED and MVPA. Solid line indicate the 90% confidence interval of the mean estimates from the Fitbit Flex and dash lines indicate equivalence zones of the GT3X+ (± 10% of the mean estimates from each cut-points)

We found strong correlations for SED estimates (r = .90, ρ = .86, all *P* < .01) between GT3X+ and Fitbit Flex (Table 3). For MVPA, the correlations between Fitbit Flex and GT3X+ were moderate across the ActiGraph cut-points applied (r = .65–.76, ρ = .69–.79, all *P* < .01). Results of one-way repeated measures ANOVA revealed that there were no significant differences in daily SED estimates between Fitbit Flex and GT3X+ (mean difference [MD] = 37 min/day*, P* = 0.21); however, the MVPA estimate from the Fitbit Flex was statistically significantly different when compared with the estimates from the GT3X+ based on three different cut-point criteria (MD = 59–77 min/day, *P* < .01) Significant differences remained only for MVPA pairwise comparisons (Table 4). Fitbit Flex significantly overestimated MVPA compared to all GT3X+ criteria by notably wide margins. The mean differences in MVPA estimates between Fitbit Flex and GT3X+ were 60 (VM3), 78 (Freedson), and 79 min/day (Troiano), respectively. The BA plots and Pitman’s Test revealed that there were no apparent bias for the agreement and variances in SED estimates (mean difference: − 37.36 min/day, limits of agreement [LOA]: − 119.73 to 45.01 min/day, R^2^ = 0.004, *P* = 0.61) between the two devices (Fig. 1). However, for MVPA, the mean differences (LOA) were − 77.67 min/day (LOA: − 126.15 to − 29.19 min/day) for Freedson, − 79.09 min/day (LOA: − 128.37 to − 29.81 min/day) Troiano, and − 59.64 (*P* < .05) VM3 cut-points, respectively. The results from the Pitman’s Test were R^2^ = 0.37 (*P* < .05) for Freedson, R^2^ = − 0.38 (*P* < .05) for Troiano, and R^2^ = − 0.16 (*P* < .05) for VM3 cut-points, suggesting that Fitbit Flex increasingly overestimates MVPA compared to GT3X+ as mean volume of MVPA increases. Results from the equivalent tests are presented in Fig. 2. The Fitbit Flex recorded equivalent estimates of SED (Mean (90% CI): 637.8 min/day (619.2–656.4)) as the GT3X+ (Mean (EZ): 600.4 min/day (540.4–660.4)). MVPA estimates from the GT3X+ (Freedson Mean (EZ): 30.1 min/day (27.1–33.1), Troiano Mean (EZ): 28.7 min/day (25.8–31.6), VM3 Mean (EZ): 48.1 min/day (43.3–52.9)) were not equivalent to the MVPA estimate from the Fitbit Flex (Mean (90% CI): 107.8 min/day (101.1–114.4)).

**Table 3:** Pearson (r) and Spearman (ρ) Correlations between Fitbit and GT3X+ SED and MVPA estimates

		Fitbit Flex	
		SED (r/ρ)	MVPA (r/ρ)
GT3X+	SED^a^ (Freedson/Troiano)	0.90^‡^ / 0.86^‡^	−0.28^†^ / -0.32^‡^
	MVPA^b^ (Freedson)	−0.24 / -0.25^†^	0.66^‡^ / 0.71^‡^
	MVPA (Troiano)	−0.22 / -0.24	0.65^‡^ / 0.69^‡^
	MVPA (VM3)	−0.31^†^ / -0.35^†^	0.76^‡^ / 0.79^‡^

^a^SED: sedentary behavior

^b^MVPA: moderate-to-vigorous physical activity

^†^*P* < .05; ^**‡**^*P* < .01;

**Table 4:** Mean differences (SE) and Mean Absolute Percent Errors of SED and MVPA between GT3X+ and Fitbit

Intensity	Comparison	Mean Difference (SE)	95% CI	*P*-value	MAPE (SD)
SED^a^ (min/day)	Freedson/Troiano - Fitbit	−37.4 (5.1)	−27.2, − 47.6	0.21	6.8% (5.5)
MVPA^b^ (min/day)	Freedson - Fitbit	−77.7 (23.9)	−88.4, −66.4	< .0001	73.0% (13.0)
	Troiano - Fitbit	−79.1 (24.3)	−89.9, −67.8	< .0001	74.3% (12.8)
	VM3 - Fitbit	−59.7 (20.6)	−70.7, 48.7	< .0001	56.6% (14.6)
	Freedson - Troiano	1.4 (4.2)	−9.4, 12.3	0.99	6.7% (5.7)
	Freedson – VM3	−18.0 (4.2)	−28.9, −7.2	< .0001	39.1% (6.5)
	Troiano – VM3	−19.5 (4.2)	−30.3, −8.6	< .0001	42.3% (18.9)

^a^*SED* sedentary behavior, ^b^*MVPA* moderate-to-vigorous physical activity, *MAPE* Mean absolute percent error

## Discussion

This study examined the accuracy of the Fitbit Flex PA monitor against a previously validated accelerometer, the ActiGraph GT3X+, for classifying SED and MVPA in free-living settings. Our results demonstrated moderate to strong relationships between the Fitbit Flex and GT3X+ monitors for SED and MVPA estimates. The Fitbit Flex provided SED estimates that were equivalent to those from the SED cut-points for the GT3X+. However, MVPA estimates from the Fitbit Flex significantly differed from and were not equivalent to MVPA estimates from the GT3X+. The observed differences show there were greater discrepancies between Fitbit Flex-determined MVPA estimates and GT3X+ cut-point criteria developed from single axis regression equations (i.e. Freedson and Troiano cut-points). Nonetheless, relative to all the GTX+ cut-points used, Fitbit markedly overestimated mean daily MVPA. Furthermore, BA plots showed these differences increased as the volume of MVPA increased, suggesting that the Fitbit Flex may overestimate MVPA in active individuals compared to the GT3X+.

Previous research has shown strong correlations for EE, step, and MVPA estimates between hip-worn Fitbit models (i.e. Fitbit One, Ultra, Zip) and ActiGraph GT3X+ [22, 23, 39, 40]. Similarly, Fitbit Flex and GT3X+ MVPA estimates have strong correlations in studies of young adult and elderly populations, with moderate correlations for LPA reported also within the elderly populations [41, 42]. Our results show the MVPA correlations between Fitbit Flex and GT3X+ estimates fall between that of these two studies. Differences in methodologies may partially explain these differences. Sushames et al. used a protocol lasting less than 24 h, with a mix of scripted PA and free-living activity [42]. Alharbi and colleagues investigated free-living activity over a 4-day period in older adults in a clinical setting [41]. Our study collected free-living data over a longer period and with a more diverse age-range of healthy adults. Thus, the longer study protocol in our investigation may better represent the relationships between Fitbit Flex and GT3X+ in free-living conditions.

Though few studies have assessed the Fitbit Flex classification estimates for SED in free-living settings, recent studies suggest the wrist-worn Fitbit Flex SED estimates will be significantly lower than those of hip-worn GT3X+. A recent study by Reid et al. demonstrated Fitbit Flex significantly underestimated SED compared to GT3X+ with a mean bias of 76.8 (minutes/day) (*p* < .05) such that increasing time spent in SED produced greater discrepancies between the devices [43]. However, Rosenberger found GT3X+ and the hip-worn Fitbit One yielded equivalent estimates of SED during a free-living protocol involving 40 participants [44]. Both GT3X+ and Fitbit One systematically underestimated SED relative to AcitPal criterion, with very similar slopes (− 0.47 and − 0.34, respectively) and mean differences (48 min/day and 34 min/day, respectively). In our study, the estimated SED between GT3X+ and Fitbit Flex was not significantly different. Furthermore, our results show a strong correlation between Fitbit Flex and GT3X+. Dominick and colleagues also reported a significant though less strong correlation between Fitbit Flex and GT3X (r = .63) though the monitors produced significantly different daily SED estimates [45]. Previous research has shown accelerometers placed at the hip demonstrate less count variability than wrist and ankle placement over a wide range of sedentary and physical activities [7]. However, though neither the Fitbit Flex nor the GT3X+ are considered gold standard for monitoring SED in free-living conditions, the level of agreement between Fitbit Flex and GT3X+ suggests monitor placement may not be the leading factor influencing the accuracy of SED estimates and merits further investigation.

To the point, defining count-based criteria for SED is inconsistent and may be operationalized to include variables such as posture, a variable not captured by the Fitbit Flex [46]. The GT3X+ has the low-frequency option, allowing the user to increase the monitor’s sensitivity to movement by lowering the frequency threshold for recording accelerations. However, based on current evidence, it is unclear whether researchers should enable the low-frequency extension feature when initializing the ActiGraph if the goal is to specifically monitor SED [47], thus we did not apply the low-frequency extension in our study.

Though the Freedson and VM3 cut-points were derived from accelerometer output using different numbers of axis (i.e. vertical axis only versus vector magnitude of three axes), research has demonstrated that the equations perform similarly compared to IC-criterion [6, 16]. Furthermore, it is possible that PA classification estimates based on triaxial monitors may yield superior PA estimates compared to vertical axis output alone. Evidence suggests the magnitude of the differences depends on the regression equation utilized and the intensity of the activity being analyzed [15]. In our study the MVPA estimates were significantly different between Freedson and Troiano cut-points compared to VM3 cut-points. Regardless, Fitbit Flex MVPA estimates were consistently significantly higher than any GT3X+ estimates.

Our results show the Fitbit Flex and GT3X+ produce very different estimates of MVPA. Specifically, Fitbit Flex overestimated mean daily MVPA by nearly an hour, or more, compared to the ActiGraph GT3X+. The hip-worn Fitbit One has overestimated MVPA compared to GT3X+, with researchers reporting mean absolute percent errors of over 60% [44]. In our study the discrepancies may be exaggerated further due to the wrist placement of the Fitbit Flex.

Recently Nelson and colleagues found Fitbit Flex overestimated the metabolic cost of walking (3.3–4.6 METs) and jogging (7.0–7.9 METs) activities compared to IC criterion [30]. However, the activities were only performed for five minutes. In our study, participants averaged nearly 30 min/day of MVPA determined by GT3X+ accelerometer. Thus, we might expect the magnitude of the discrepancy in MVPA estimates between GT3X+ and Fitbit Flex to be much greater. In support of this explanation in our analysis of the BA plots of Fitbit Flex and GT3X+ MVPA, each data plot was below zero, indicating the Fitbit Flex overestimated mean daily MVPA for each participant. We also observed a negative slope for the fit line, suggesting that this discrepancy tends to increase as total mean daily MVPA volume increases. Other research has found similar systematic bias for Fitbit Flex step estimates, but not for EE estimates [28, 41, 42].

The strengths of this investigation include the length of the free-living protocol, the wide age range represented in the participant sample, and high number of valid wear days. Only two previous studies have investigated the wrist-worn Fitbit Flex in a protocol lasting at least seven days and those studies only included 22 or fewer participants between ages 19–37 [43, 45]. In addition, our investigation included a wrist-worn consumer-based accelerometer-based monitor, which are more popular [21] than hip worn models and may potentially increase compliance in future research studies. In contrast to previous investigations that have utilized only single cut-point criteria for estimating SED and MVPA from ActiGraph accelerometers [23, 40, 44], we evaluated the validity of the Fitbit Flex against the GT3X+ when applying three different previously validated cut-points indicating our results are not limited by the use of a single cut-point. Lastly, finding from this study can be applicable to studies using more recent models of Fitbit (i.e., Flex 2) because there was high inter-monitor reliability as evidenced by a high intra-correlation coefficient (ICC) value of 0.91 (data not presented in detail herein) when we tested the inter-monitor reliability between Fitbit Flex and Fitbit Flex 2 in a separate study.

Certain limitations of this study must be considered when interpreting our results. Fitbit does not currently have a wear time validation mechanism per se, though other researchers have applied typical validation approaches to minute-by-minute Fitbit data where 60 consecutive minutes of no PA during waking hours are assumed to be non-wear time [45]. Thus, it is not possible to truly know if such occurrences are due to non-wear time or extensive SED. Limitations of using ActiGraph for assessing SED have been reported; however, previous research has shown acceptable estimates of SED compared to ActivPAL and IC criterion [30]. Another limitation was that there was no true gold-standard method used to evaluate the validity of the Fitbit Flex, thus no inference can be made about the criterion validity of the Fitbit Flex. Lastly, both Fitbit Flex and GT3X+ are not completely waterproof therefore we were unable to capture activities such as swimming or bathing for this analysis.

## Conclusions

In conclusion, our data suggest that the Fitbit Flex and GT3X+ were statistically equivalent to one another in assessing SED, but not MVPA; the MVPA estimates were significantly overestimated by the Fitbit Flex. On-going population surveillance will benefit from improved objective monitoring options that will maximize subject compliance and data accuracy. Improving the accuracy of MVPA monitoring is paramount to increasing population adherence to the Physical Activity Guidelines for Americans. Consumer-based PA monitors, such as the Fitbit Flex, show promise for promoting PA adherence to the general public by allowing individuals to self-monitor daily PA. However, if the Fitbit Flex overestimates MVPA, this may reduce the likelihood that an individual would meet the minimum recommended MVPA. Further research is needed to investigate the accuracy and precision of Fitbit Flex PA classification estimates in free-living settings.

## Abbreviations

BA plot: Bland-Altman plot
BMI: Body mass index
EE: Energy expenditure
EZ: Equivalence zone
MAPE: Mean absolute percent error
MVPA: Moderate-to-vigorous physical activity
PA: Physical activity
SED: Sedentary behavior
